# AMP-activated protein kinase deficiency reduces ozone-induced lung injury and oxidative stress in mice

**DOI:** 10.1186/1465-9921-12-64

**Published:** 2011-05-19

**Authors:** Sébastien Hulo, Hélène Tiesset, Steve Lancel, J Louis EdméJeanjledme@univ-lille2.fr, Benoit Viollet, Annie Sobaszek, Rémi Nevière

**Affiliations:** 1 Univ Lille Nord de France, F-59 000 Lille, France; 2 UDSL, EA 4483, F-59 000 Lille, France; 3 INSERM U 1019, F-59 000 Lille, France; 4 UDSL, EA 4484, F-59 000 Lille, France; 5 INSERM, U1016, Institut Cochin, Paris, France; 6 Cnrs, UMR8104, Paris, France; 7 Univ Paris Descartes, Paris, France

## Abstract

**Background:**

Acute ozone exposure causes lung oxidative stress and inflammation leading to lung injury. At least one mechanism underlying the lung toxicity of ozone involves excessive production of reactive oxygen and nitrogen intermediates such as peroxynitrite. In addition and beyond its major prooxidant properties, peroxynitrite may nitrate tyrosine residues altering phosphorylation of many protein kinases involved in cell signalling. It was recently proposed that peroxynitrite activates 5'-AMP-activated kinase (AMPK), which regulates metabolic pathways and the response to cell stress. AMPK activation as a consequence of ozone exposure has not been previously evaluated. First, we tested whether acute ozone exposure in mice would impair alveolar fluid clearance, increase lung tissue peroxynitrite production and activate AMPK. Second, we tested whether loss of AMP-activated protein kinase alpha1 subunit in mouse would prevent enhanced oxidative stress and lung injury induced by ozone exposure.

**Methods:**

Control and AMPKα1 deficient mice were exposed to ozone at a concentration of 2.0 ppm for 3 h in glass cages. Evaluation was performed 24 h after ozone exposure. Alveolar fluid clearance (AFC) was evaluated using fluorescein isothiocyanate tagged albumin. Differential cell counts, total protein levels, cytokine concentrations, myeloperoxidase activity and markers of oxidative stress, i.e. malondialdehyde and peroxynitrite, were determined in bronchoalveolar lavage (BAL) and lung homogenates (LH). Levels of AMPK-Thr^172 ^phosphorylation and basolateral membrane Na(+)-K(+)-ATPase abundance were determined by Western blot.

**Results:**

In control mice, ozone exposure induced lung inflammation as evidence by increased leukocyte count, protein concentration in BAL and myeloperoxidase activity, pro-inflammatory cytokine levels in LH. Increases in peroxynitrite levels (3 vs 4.4 nM, p = 0.02) and malondialdehyde concentrations (110 vs 230 μmole/g wet tissue) were detected in LH obtained from ozone-exposed control mice. Ozone exposure consistently increased phosphorylated AMPK-Thr^172 ^to total AMPK ratio by 80% in control mice. Ozone exposure causes increases in AFC and basolateral membrane Na(+)-K(+)-ATPase abundance in control mice which did not occur in AMPKα1 deficient mice.

**Conclusions:**

Our results collectively suggest that AMPK activation participates in ozone-induced increases in AFC, inflammation and oxidative stress. Further studies are needed to understand how the AMPK pathway may provide a novel approach for the prevention of ozone-induced lung injury.

## Background

Ambient ozone is a secondary pollutant formed through a complex series of photochemical reactions involving nitrogen oxides and sunlight. Ozone inhalation causes airway inflammation, direct epithelial injury and decreased lung function in both animal and human studies [1]. Ozone is highly reactive and oxidizes proteins and lipids in the fluid-lining compartment of the lung, which initiates inflammation processes and increased lung permeability. At least one mechanism underlying the lung toxicity of ozone involves excessive production by airway epithelial cells and activated lung immune cells of cytotoxic mediators including pro-inflammatory cytokines, reactive oxygen and nitrogen intermediates such as peroxynitrite [2,3].

Peroxynitrite is an extremely powerful and cytotoxic oxidant, which not only oxidizes lipids, nucleic acids, and protein SH-groups, but also nitrates aromatic amino acid protein residues. Via nitration of tyrosine residues, peroxynitrite alters the function of many proteins, such as superoxide dismutase, prostacyclin synthase PGI_2_, Ca^2+^-ATPase, glutathione s-transferase, as well as transcriptional factors [4,5]. Observations that peroxynitrite induces nitration of tyrosine residues and also alters tyrosine phosphorylation have focused attention on phosphorylation cascades, as phosphorylation of mitogen-activated protein kinases modulate many cell signaling processes [4,6]. Moreover, it was recently proposed that peroxynitrite strongly increases phosphorylation of constitutive nitric oxide synthase via 5'-AMP-activated kinase activation, which results in enhanced production of reactive oxygen species contributing to a further oxidative damage [4].

5'-AMP-activated protein kinase (AMPK) is a serine threonine kinase that is highly conserved through evolution. As a cellular metabolic sensor, AMPK acts on a wide variety of substrates and cellular pathways, including the regulation of metabolic pathways (e.g., glycolysis, fatty acid synthesis and oxidation, cellular glucose uptake, and cholesterol synthesis), apoptosis and the cell cycle. It is becoming increasingly recognized that changes in AMPK activation are also implicated in several signaling pathways involved in nitric oxide production, sensing and responding to oxidative stress, and inflammation [7-9]. Downstream effects of AMPK activation may differ depending upon tissue stress intensity and metabolic needs. In peripheral organs such as the heart, activation of AMPK during ischemia reperfusion reduces damage [10]. In the brain, activation of AMPK during low-energy states such as ischemia is detrimental [11]. In the lung, AMPK activation following hypoxic challenge results in pulmonary vasoconstriction and transepithelial Na^+ ^transport inhibition [12]. Hence, biological consequences of stress-mediated AMPK activation are complex and remain highly variable.

Alterations in AMPK activation as a consequence of ozone exposure have not been previously evaluated. It is likely that production of peroxynitrite induced by ozone exposure may activate AMPK, which in turn could modulate transepithelial ion transport, oxidative stress and inflammation. Our objectives were twofold. First, we tested whether acute ozone exposure in mice would impair alveolar fluid clearance, increase lung tissue peroxynitrite production and activate AMPK. Second, we tested whether loss of AMP-activated protein kinase alpha1 subunit in mouse would prevent enhanced oxidative stress and lung injury induced by ozone exposure.

## Methods

### Animal used

Control and AMPKα1 KO male mice (129/Sv and C57BL/6 mixed background) were studied at 20-24 wk of age. Animals were housed under controlled temperature and lighting with free access to water and standard mouse chow diet. All procedures were performed in accordance with the principles and guidelines established by the European Convention for the Protection of Laboratory Animals. All animal studies were approved by the Institutional Review Board of the National Institute (Agreement number 59-350146, Direction départementale de Protection des Populations DDPP, Nord, France).

### Ozone exposure

Exposure to ozone [2 parts/million (ppm) for 3 h] or filtered room air in conscious mice was conducted in a stainless steel chamber with a Plexiglas door (~2.5 liters in volume). Ozone was generated by passing dry 100% oxygen through ultraviolet light and mixing it with filtered room air in the chamber. Chamber atmosphere was drawn continuously via a sampling port, and ozone concentration was measured by an ozone chemiluminescent analyzer (Model 49I; Thermo-environmental Instruments, Franklin, Massachussetts, USA). Chamber air temperature and relative humidity were maintained constant throughout experiments. Mice were then allowed to recover in ambient air for 24 h. Mice were then euthanized with an i.p. injection of a mixture of ketamine and xylazine (100 mg/kg and 20 mg/kg respectively).

### Bronchoalveolar lavage (BAL)

Mice were sacrificed, and the trachea was cannulated with a 20-gauge catheter. BAL was performed twice with 0.8 ml of ice-cold PBS (pH 7.4) each. At least 1 mL of injected volume was recovered in all mice. The BAL fluid was spun at 800 g for 5 min at 4°C, and supernatant was collected for the measurement of total proteins. Total proteins from cell free supernatant of the BAL fluids were assessed using the Bradford Assay (BioRad, CA). Pelleted cells were harvested, and red cells were lysed, then washed and resuspended in cold PBS. Total cells were enumerated by counting on a hemocytometer. For differential cell counting, cells were spun onto glass slides, air-dried, fixed in ethanol, and stained with Diff-Quick reagents (Baxter Scientific, Miami, FL). The number of macrophages, neutrophils and lymphocytes was counted based on morphology.

### Myeloperoxidase activity, malondialdehyde level and peroxynitrite content in lung homogenates

Animals were euthanized and perfused with cold saline via the heart. The left lobes were removed and homogenized in 1 ml of PBS containing complete protease inhibitor cocktail (Sigma, St. Louis, MO). Then, the samples were centrifuged for 10 min at 800 g. Supernatants were filtered and kept in -70°C until used. Myeloperoxidase (MPO) activity, an indicator of polymorphonuclear leukocyte (PMN) accumulation, was determined as previously described [13]. Briefly, following lung homogenate preparation, myeloperoxidase activity was determined by measuring hydrogen peroxide-dependent oxidation of 3,3',5,5'-tetramethylbenzidine. The rate of change in absorbance was measured spectrophotometrically at 650 nm. Myeloperoxidase activity was defined as the quantity of enzyme degrading 1 μmol of peroxide min^-1 ^at 37°C and was expressed in units per gram weight of wet tissue. Malondialdehyde (MDA) level was utilized to quantify the lipid peroxidation in tissues as previously described [14]. Briefly, following lung homogenate preparation, MDA content was based on the reaction of MDA with thiobarbituric acid at 90-100°C. Samples were then heated for 1 h at 95°C and centrifuged at 3000 g for 10 min. The absorbance of the supernatant was measured by spectrophotometry at 532 nm using 1,1,3,3-tetramethoxypropane as an external standard. MDA was expressed in micromole per g weight of wet tissue. The formation of peroxynitrite was estimated by means of peroxynitrite-dependent oxidation of dihydrorhodamine-123 (DHR123, Molecular Probes, Eugene, Oregon) to rhodamine as previously described. This method is an indirect measurement of peroxynitrite production since other oxidant species can oxidize dihydrorhodamine-123 [15]. Briefly, duplicate aliquots of lung homogenate supernatants incubated with 5 μM DHR123 were taken for rhodamine fluorescence evaluation using a fluorometer at an excitation wavelength of 500 nm and an emission wavelength of 530 nm. A fluorescence standard curve was obtained with authentic rhodamine and results were expressed in nmol/L.

### Multiplex for detection of cytokines

Lung homogenate supernatants were assayed for a panel of cytokines and chemokines (IFN-γ, IL-1 α, IL-1β, TNF-α, IL-6, IL-10, IL-2, IL-4, IL-5, IL-7, IL-9, IL-10, IL-12p40/p70, IL-13, IL-15, IL-17, IP-10, MCP-2, G-CSF, MIP-1α, GM-CSF, and RANTES) using a multiplex-assay (xMAP Luminex Bioplex 200, Luminex Corp, Biorad, France) according to the manufacturer's instructions. All samples were assayed in duplicate and results were calculated as pg/mL.

### Alveolar fluid clearance

After sacrifice, the trachea was cannulated by using a 20-gauge catheter. The lungs were then inflated with 7 cm H2O continuous airway pressure with 100% oxygen throughout the experiment. An infrared lamp placed above the body maintained body temperature at 37°C. The lungs were instilled with Ringer lactate containing 5% bovine serum albumin and fluorescein isothiocyanate-tagged albumin as an alveolar protein tracer. Osmolarity of the instillate was adjusted to 340 mosM, which we have shown to be isosmolar with mouse plasma. Alveolar fluid clearance (AFC) was measured over 15 min. As in previous studies, AFC was determined by measuring the increase in the final fluorescence intensity of the alveolar protein tracer compared with the initial instilled tracer protein fluorescence intensity [16,17]. AFC was calculated as AFC = 1-(C1/C2), where C1 is the fluorescence intensity of the initial instilled tracer protein and C2 is the final total tracer protein fluorescence intensity in the alveolar fluid.

### Basolateral cell membrane isolation

Pieces of peripheral lung tissue were collected from each lobe and homogenized to obtain whole cell lysates (each piece approximately represented a cube of 2 millimeters side). Cells were prepared by addition of lysis buffer and centrifugation at 14,000 g to eliminate the insoluble material. Basolateral membranes were prepared using ultracentrifugation as previously described [18]. Peripheral lung tissue was homogenized and centrifuged twice to discard the nuclear and mitochondrial pellet. Supernatant was centrifuged at 48,000 g for 30 min, and the basolateral membrane fraction was recovered after the membrane pellet was centrifuged in a 16% Percoll gradient at 48,000 g for 30 min.

### Western blot analysis

Proteins from cell lysates and basolateral membrane fractions were resolved by 10% SDS-PAGE and analyzed by immunoblotting. Mouse monoclonal antibodies anti total AMPK-α (Cell Signaling, France) and rabbit monoclonal antibodies anti-phospho-AMPK-α at Thr172 (Cell Signaling, France) were used for cell lysate immunoblotting. Mouse monoclonal antibody anti-Na,K-ATPase alpha_1 _(Upstate Biotechnology, Lake Placid, NY) and rabbit polyclonal antibody anti-GLUT1 (Millipore, France) were used for basolateral membrane fractions immunoblotting, respectively. AMPK activation was assessed as the ratio of phospho-AMPK to total AMPK. GLUT1 was measured by immunoblotting as a loading control for plasma membrane protein. The amount of Na,K-ATPase at the plasma membrane was normalized to GLUT1.

### Statistical analysis

All statistical analyses were performed using GraphPad Prism 5.0 (GraphPad Software Inc., San Diego, CA). When a significant difference was found, we examined between-group differences using a sequentially rejective Bonferroni procedure. After applying Bonferroni's correction, the level of statistical significance was set at *p *< 0.05 for comparisons with control. Experimental results were expressed as mean ± S.D.

## Results

### Ozone exposure induces lung inflammatory

**Figure 1:**
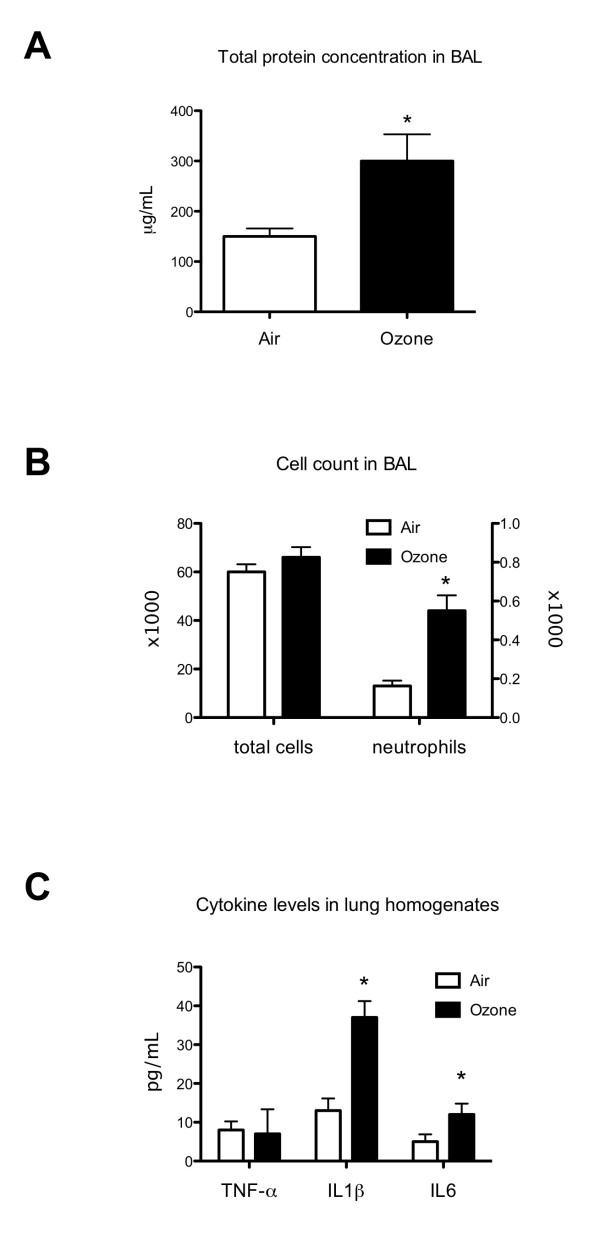
**Effects of ozone exposure on lung inflammation in control mice**. Total protein concentration (A), total cell count and neutrophil count (% × 1 000) (B) in BAL fluid and pro-inflammatory cytokine levels (C) in lung homogenates were measured as markers of pulmonary inflammation. Ozone-exposed groups were exposed to air containing 2 ppm ozone continuously for 3 h. Parameters were evaluated 24 h after exposure. Data are presented as means ± SD. *p < 0.05, when compared with non-exposed mice (n = 10 in control group and n = 8 in ozone-exposed group). BAL: bronchoalveolar lavage; TNF-α: tumor necrosis factor alpha; IL1β: interleukin 1 beta; IL6: interleukin 6

In the first part of our study, we observed the impact of acute ozone exposure on lung inflammation. We therefore evaluated cell recruitment and total protein level in the BAL. We found a significant increase in proportion of neutrophils in the ozone-exposed mice BAL (Figure 1). The recruitment of neutrophils observed in mice BAL has been confirmed in lung homogenates by measurement of MPO activity which was significantly higher in ozone-exposed mice than in control mice (p = 0.037) (Table 1). The total protein was also higher in ozone-exposed mice BAL than in control mice. We also showed a significant increase in major pro-inflammatory cytokine levels such as IL-6 and IL-1β in ozone-exposed mice lung homogenates (Figure 1).

**Table 1:** MDA levels, MPO activity and peroxynitrite levels in lung homogenates WT or AMPKα1 deficient mice

	control mice		AMPKα1 deficient mice		
			
	Air	Ozone	Air	Ozone	
MDA (μmol/g wet tissue)	110 +/- 14.1*	230 +/- 10*	160 +/- 13	147 +/- 12.6	*p < 0.0001

MPO (U/g wet tissue)	0.13 +/- 0.03*	0.27 +/- 0.04*	0.13 +/- 0.02	0.16 +/- 0.06	*p = 0.04

Peroxynitrite (nmol/L)	3.0 +/- 0.2*	4.4 +/- 1.0*	2.8 +/- 0.5	2.8 +/- 0.4	*p = 0.03

Mice were exposed to air with or without ozone at 2 ppm for 3 h. Parameters were evaluated 24 h after exposure. Malondialdehyde (MDA) was expressed as micromole per g weight of wet tissue; Myeloperoxidase activity (MPO) was expressed in units per gram weight of wet tissue. Peroxynitrite was expressed as nmol/L. Data are presented as means ± SD. *: p < 0.05; μmol/g wet tissue: micromole per g weight of wet tissue; U/g wet tissue: units per gram weight of wet tissue

### Ozone exposure increases lung oxidative stress

There were significant increases in oxidation of DHR123 (p = 0.02) and MDA concentration (p < 0.0001) in ozone-exposed mice lung homogenates (Table 1).

### Ozone exposure increases AMPK phosphorylation

**Figure 2:**
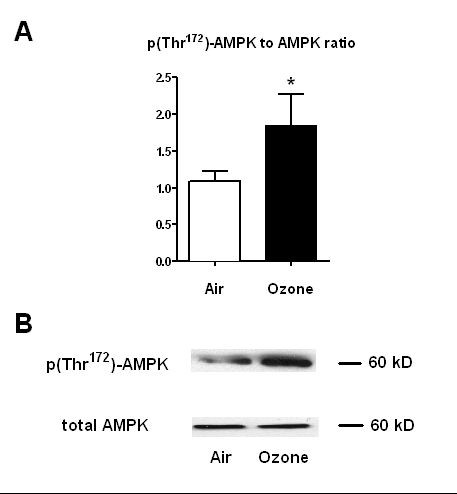
**P(Thr172)-AMPK to total AMPK ratio in control mice lung homogenates**. P(Thr^172^)-AMPK to total AMPK ratio (A) and Western Blot analysis (B) were performed in control mice lung homogenates. Mice were exposed or not to ozone at 2 ppm for 3 h. Parameters were evaluated 24 h after exposure. Data are presented as means ± SD. *: p < 0.05; (n = 6 in each group). AMPK: adenosine monophosphate activated protein kinase

As we observed a peroxynitrite increase in ozone-exposed mice lung homogenates, we next evaluated AMPK phosphorylation. There was a strong increase of phospho-AMPK to total AMPK ratio in ozone-exposed mice lung homogenates compared with the unexposed group (p = 0.04) (Figure 2).

### Ozone exposure increases alveolar fluid clearance

**Figure 3:**
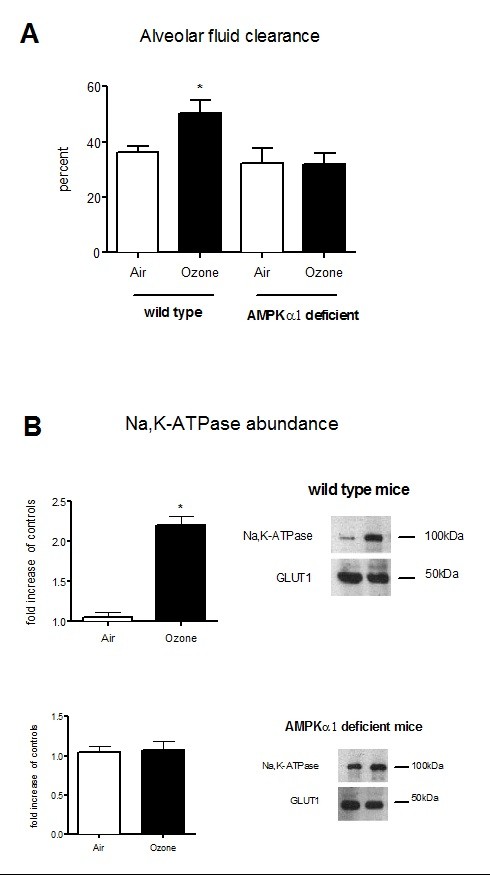
**Alveolar fluid clearance and basolateral membrane Na,K-ATPase abundance in WT or AMPKα1 deficient mice**. (A) Alveolar fluid clearance (%) in wild type or AMPKα1 deficient mice exposed (n = 3) or not (n = 4) to ozone at 2 ppm for 3 h. (B) Western Blot analysis of Na,K-ATPase expression at the plasma membrane in wild type or AMPKα1 deficient mice exposed (n = 3) or not (n = 4) to ozone at 2 ppm for 3 h. The amount of Na,K-ATPase was normalized to GLUT1. Results were expressed in fold increased of control. Mice were exposed to air with or without ozone at 2 ppm for 3 h. Parameters were evaluated 24 h after exposure; *: p < 0.05; GLUT1: glucose transporter 1.

As we observed an increase in the total protein in BAL of mice exposed to ozone, which indicated an increase in crude epithelial permeability, we explored the effects of ozone on the alveolar fluid clearance increase. So, we hypothesized that activation of Na,K-ATPase contributes to the increase in clearance of alveolar fluid. There was a significant increase of alveolar fluid clearance in ozone-exposed control mice (p = 0.05). We then wanted to know if the Na,K-ATPase was partly responsible for the increased alveolar fluid clearance. Basolateral membrane Na,K-ATPase abundance normalized to GLUT1was twofold higher in ozone-exposed control mice (Figure 3).

### AMPKα1 deficient mice are protected from ozone lung injury

There were no changes in MPO activity, MDA concentration and peroxynitrite levels in AMPKα1 deficient mice after ozone exposure (Table 1). Alveolar fluid clearance did not change significantly in AMPKα1 deficient mice after ozone exposure (Figure 3). The basolateral membrane Na,K-ATPase abundance was not different between the exposed and unexposed group in AMPKα1 deficient mice.

## Discussion

Because AMPK has been shown to modulate airway transepithelial ion transport, inflammation and oxidative stress, we tested the hypothesis that AMPK deficiency would prevent ozone-induced lung injury in mice. Important findings of this study revealed that acute ozone exposure increase lung fluid clearance, leukocyte infiltration and oxidative stress and that ozone-induced lung injury was largely prevented in AMPK alpha1 subunit deficient mice.

Acute inhalation of ozone was found to cause structural alterations in the lung including disruption of the alveolar epithelial barrier, followed by alveolar epithelial type II cell hypertrophy and hyperplasia. Recruitment of inflammatory cells into the lung following O3 exposure presents another risk of tissue damage through the release of toxic mediators by activated macrophages and neutrophils inflammatory cells, including cytokines, reactive oxygen and nitrogen species and proteolytic enzymes [1]. Following ozone inhalation, TNF-α levels rapidly peaked, predominantly in alveolar macrophages and has been implicated in lung injury by stimulating the production of cytotoxic inflammatory mediators such as cytokines and chemokines. In rodents, many of the pulmonary manifestations of acute O3 exposure have been attributed to the effects of these cytokines and chemokines, as interruption of IL-1, IL-6, signaling significantly diminishes O3-induced neutrophil emigration to the airspaces and/or epithelial cell injury. Twenty-four hours after exposure early TNF-α peak concentration was not detected, whereas concentrations of IL-1 and IL-6 increased in BAL of ozone-exposed mice. This is consistent with previous studies indicating that cytokine secretion profiles are related to compartmentalization and migration of different inflammatory cell-types during the development of ozone injury [1-3]. In addition, characteristics of recruitment of inflammatory cells in mice lung following ozone exposure have been shown varying with ozone concentration, exposure duration, age and strains [1]. In these regards, C57bl6/129sv mice used in our model are strong responders for the lung response to O3 [19] and seem to be little affected by age in term of O3 response [20]. Consistently, our model of acute ozone exposure (3 h, 2 ppm) elicited alveolar neutrophil recruitment and increases in biomarkers of lung tissue inflammation and oxidative stress. Expression of concentrations of biomarkers per gram of wet lung in this study would have caused us to underestimate increases in MDA, MPO and peroxynitrite levels, as O3 exposure likely induced edema formation leading to increases in lung wet weight, which we used as a normalization parameter.

Fluid and solute resorption from the alveolus is critical in clearing fluid from lungs [21]. Active alveolar epithelial Na^+ ^ion transport is well established as the primary mechanism of fluid clearance from distal airspaces [22]. In the current model of fluid balance in the distal lung, Na^+ ^ions enter alveolar type II epithelial cells at the apical surface primarily through amiloride-sensitive sodium channels and are pumped out on the basolateral surface by Na,K-ATPase. In conditions of lung inflammation, such as ozone exposure, active Na^+ ^transport and alveolar fluid clearance may be stimulated leading to increased ability of the lungs to clear edema. Indeed, a large variety of endogenous mediators, which are released by the lung in response to ozone exposure, may be responsible for the stimulation of alveolar liquid clearance. For example, increased TNF-alpha production has been consistently shown to stimulate alveolar fluid transport [23]. Likewise, authentic nitric oxide and nitrogen intermediates have been shown to modulate active Na^+ ^transport in alveolar type 2 epithelial cells, and both stimulation [24] and inhibition [25] of transport characteristics have been reported. Hence, it may be hypothesized that alveolar fluid clearance increase observed in our ozone exposure model may be the result of ozone-induced proinflammatory cytokine and peroxynitrite increases.

Concomitant changes in permeability, oxidative stress and recruitment of inflammatory cells in the lung following ozone exposure have been shown varying with ozone concentration, exposure duration, and animal species [1]. The exact mechanism influencing the severity of ozone-induced pulmonary reaction and molecules involved in the modulation of this response are yet to be fully determined. Proposed mechanisms include the regulation of the oxidant anti-oxidant balance through functionally relevant post-translational protein modification. In this context, peroxynitrite can play an important role in this balance through the nitration of tyrosine residues that alters the function of many proteins, such as superoxide dismutase and NADPH oxidase [6]. In addition, via increased c-Src, phosphatidylinositol 3-kinase or protein kinase C activities, peroxynitrite may also activate AMPK, which has been shown to alter cell response to oxidative stress [26-28]. In our study, we confirmed that increased lung peroxynitrite production was associated with AMPK phosphorylation in ozone-exposed mice. Importantly, AMPK alpha1 subnunit deficient mice were protected against ozone-induced lung injury, suggesting that AMPK activation was detrimental.

AMPK is a heterotrimeric Ser/Thr kinase composed of a catalytic α subunit and regulatory β and γ subunits. The α1 and α2 subunit isoforms have ~90% homology in their N-terminal catalytic domains and ~60% homology in their C-terminal domains, suggesting that they may have distinct downstream targets. For example, AMPKα2 deletion results in mild insulin-resistance and impaired glucose tolerance, whereas deletion of AMPKα1 has no detectable effects on metabolic phenotype. In addition and beyond the regulation of energy homeostasis, AMPK also exerts non-metabolic functions, such as maintenance of cell polarity and normal cell division, or control of cell growth and survival [29]. In lung cells, the α1 catalytic subunit of AMPK is predominantly expressed and its pharmacological activation leads to inhibition of Na^+ ^transport processes across lung epithelium [30]. It has been previously reported that AMPK activation inhibits Na^+ ^transport processes across lung epithelium via PKCzeta activation and Na,K-ATPase endocytosis, whereas inhibition of AMPK by compound C enhances Na^+ ^transport [31]. In our model, lung AMPK phosphorylation observed in control mice exposed to ozone was not associated with expected inhibition of Na^+ ^transport processes across lung epithelium and Na,K-ATPase endocytosis related to AMPK activation. Instead, ozone exposure induced increases in alveolar fluid clearance and the presence of Na,K-ATPase at the plasma membrane. Yet, increases in alveolar fluid clearance and plasma membrane Na,K-ATPase abundance were not observed in AMPKα1 deficient mice exposed to ozone, suggesting that AMPK activation modulates mechanisms involved in Na^+ ^transport processes across lung epithelium independent of Na,K-ATPase activity.

Hence, we next reasoned that protection against ozone-induced lung injury in AMPKα1 deficient mice was related to the reduction of oxidative stress and leukocyte lung infiltration. Under conditions of cell stress, the actions of AMPK are mainly dependent on cell type and metabolism [32]. AMPK activation is beneficial in peripheral tissues, such as the ischemic heart [10]. However, AMPK activation may exacerbate brain oxidative stress and injury whereas its pharmacological inhibition provided neuroprotection in the ischemic and reperfused brain [33,34]. It has been proposed that in cells lacking key glycolytic enzymes and/or with limited ability to store nutrients such as neurons, AMPK activation leads to acidosis, enhanced oxidative stress and cell apoptosis [35]. It is, hence, our contention that in lung epithelial cells, which have low nutrient storage capacity, AMPK activation may be detrimental and that defect in AMPKα1 may be protective by limiting ozone-induced lung oxidative stress. Another common feature of the host response to ozone is neutrophilic inflammation and up-regulation of proinflammatory cytokines. Recent evidence suggests that AMPK participates in the modulation of the inflammatory response. For example, it has been reported that pharmacological activation of AMPK has anti-inflammatory effects [36], whereas AMPKα1 deficient mice display normal immune responses. As neutrophilic recruitment is mainly redox-sensitive in the acute ozone-exposed lung, we speculated that reduced lung leukocyte sequestration was related to the decreased oxidative stress in AMPKα1 deficient mice.

## Conclusion

Our results collectively suggest that activation of AMPK is detrimental in ozone-induced lung injury. The use of AMPK α1 knockout mice clearly shown that AMPK activation participates in ozone-induced increases in lung fluid clearance, leukocyte infiltration and oxidative stress. Further studies are needed to delimit the role of AMPK pharmacological inhibition and to demonstrate that manipulation of the AMPK pathway may provide a novel approach for the prevention of ozone-induced lung injury.

## Abbreviations

AFC: Alveolar fluid clearance; AMPK: 5'-AMP-activated protein kinase; BAL: bronchoalveolar lavage; DHR123: dihydrorhodamine-123; G-CSF: granulocyte colonystimulating factor; GLUT: glucose transporter; GM-CSF: granulocyte-macrophage colony stimulating factor; IFN: interferon; IL: interleukin; MCP: monocyte chemotactic protein; MDA: malondialdehyde; MIP: macrophage inflammatory protein; MPO: myeloperoxidase; NADPH oxidase: nicotinamide adenine dinucleotide phosphate oxidase; PKC: protein kinase C; PMN: polymorphonuclear leukocyte; ppm: parts/million; RANTES: Regulated on Activation Normal T Cell Expressed and Secreted; SDS-PAGE: Sodium Dodecyl Sulfate-Polyacrylamide Gel Electrophoresis; TNF: tumor necrosis factor

## Competing interests

The authors declare that they have no competing interests.

## Authors' contributions

All authors have read and approved the final manuscript.

SH was responsible for carrying out the experiments, for data analysis, and for drafted this manuscript; HT was responsible for the alveolar fluid clearance study; SL and RN oversaw the animal experiments, instructed SH in his implementation; RN, SL, JLE and AS assisted in the experimental design and the data analysis and interpretation. BV is expert in AMPK α1 knockout mice and has provided AMPK α1 knockout mice. All authors contributed to the drafting and revisions of the manuscript.
